# Chromatin Modifications Orchestrate DNA Repair Pathway Choice: Epigenetics Strategies for Enhancing CRISPR–Cas9‐Mediated Knock‐In Efficiency

**DOI:** 10.1111/cpr.70285

**Published:** 2026-10-09

**Authors:** Xin Chen, Shan Qin, Ce Yang, Zhiyao Xing, Chong Liu, Xingchen Zhang, Xi Wu, Xueren Li, Yanhua Gong, Zichuan Liu

**Affiliations:** ^1^ School of Disaster and Emergency Medicine, Faculty of Medicine Tianjin University Tianjin China; ^2^ Tianjin University and Health‐Biotech United Group Joint Laboratory of Innovative Drug Development and Translational Medicine, School of Pharmaceutical Science and Technology, Faculty of Medicine Tianjin University Tianjin China; ^3^ State Key Laboratory of Synthetic Biology, Frontiers Research Institute for Synthetic Biology Tianjin University Tianjin China; ^4^ Department of Respiratory Medicine Tianjin Jinnan Hospital Tianjin China; ^5^ Department of Respiratory Medicine, Jinnan Hospital Tianjin University Tianjin China

## Abstract

CRISPR–Cas9‐mediated precise gene knock‐in relies on homology‐directed repair (HDR) at low frequencies in most mammalian cells, limiting precise gene editing in both research and therapeutic applications. Although multiple strategies have been developed to increase knock‐in efficiency, their application can be restricted by technical complexity, limited applicability, or potential safety concerns. In this review, we propose a shift in focus from conventional strategies toward epigenetic methods to increase knock‐in efficiency. It has been proved that histone modifications such as H3K36me3, H3K9me3, H4K20me2, and H2AK15ub are involved in DNA damage signallings and the recruitment of key HDR and non‐homologous end joining (NHEJ) factors. We further discuss conceptual strategies for transient chromatin modulation to bias repair pathway choice and highlight associated technical challenges and safety considerations. Together, these findings and insights highlight the essential role of histone modification in shaping chromatin states and orchestrating DNA repair dynamics. Accordingly, epigenetic regulation emerges as a compelling strategy to facilitate high‐fidelity gene knock‐in, offering valuable mechanistic clues and actionable epigenetic targets to gene editing for basic research and therapeutic translation.

## Graphical abstract

Histone modifications at CRISPR–Cas9‐induced DNA double‐strand breaks shape chromatin states and regulate DNA repair pathway choice. Transient epigenetic modulation may provide a promising strategy to enhance CRISPR–Cas9‐mediated gene knock‐in.

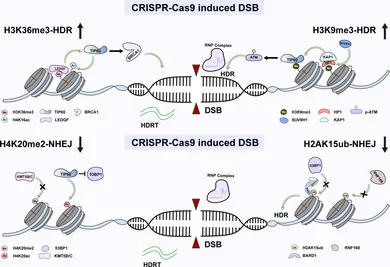

## 1 Introduction

With the rapid advances in molecular biology and protein structural genomics, gene editing technologies have sparked a new wave of innovation. These tools enable precise manipulation of genomic DNA, allowing systematic exploration of gene deletion, insertion, mutation, and variation across diverse organisms, thereby elucidating gene function at unprecedented resolution [1]. As a result, gene editing has profoundly reshaped modern cell biology, molecular biology, and genetics. Importantly, gene editing technologies support direct genetic manipulation at the cellular and even embryonic levels, greatly simplifying the construction of disease models. These advances have accelerated progress in tumour biology and epigenetics, but have also opened new therapeutic opportunities for monogenic disorders, autoimmune diseases, and cancer [2, 3, 4, 5].

Among current gene editing platforms, the CRISPR–Cas9 system has emerged as the dominant technology because of its simplicity, programmability, and scalability [6]. Unlike earlier nuclease platforms, CRISPR–Cas9 uses single‐guide RNAs (sgRNAs) to target recognition, avoiding the requirement for complex protein engineering and enabling efficient multiplex targeting and high‐throughput screening [1, 7]. In parallel, gene integration strategies have evolved from random retroviral or lentiviral transduction toward site‐specific knock‐in approaches enabled by CRISPR‐based genome editing [8, 9, 10]. Gene knock‐in technologies enable the precise insertion of exogenous DNA at defined genomic loci and have broad applications, including real‐time monitoring of gene expression and protein dynamics via reporter or epitope tagging [11], the generation of disease‐relevant alleles for mechanistic studies [12, 13, 14], and enhancement of cell‐based therapies through precise genetic modifications in stem or immune cells [15, 16, 17, 18].

Despite these advances, the broad application of gene knock**‐**in remains limited by intrinsically low efficiency. Precise CRISPR–Cas9‐mediated integration largely depends on HDR, which is predominantly active during the S/G2 phases of the cell cycle [19]. In contrast, NHEJ functions throughout the cell cycle and therefore dominates the repair of DSBs [20], resulting in a narrow temporal window for precise gene editing. Current strategies to increase knock‐in efficiency primarily focus on suppressing competing repair pathways such as NHEJ or microhomology‐mediated end joining (MMEJ), synchronizing or arresting cells in the S/G2 phase, and engineering components of the CRISPR–Cas9 system. Pharmacological or genetic inhibition of NHEJ factors can substantially increase knock‐in efficiency [16, 21, 22]; however, sustained suppression of NHEJ has raised concerns regarding genome stability and long‐term safety [23]. Similarly, manipulation of cell‐cycle checkpoints may promote HDR but can perturb normal cellular physiology [24, 25]. In addition, extensive optimization efforts—including sgRNA engineering [26, 27, 28], modification of homologous recombination donor templates (HDRTs) [29, 30, 31, 32], and Cas9 protein engineering have been reported to improve knock‐in efficiency [33, 34, 35].

More recently, accumulating evidence suggests that the modulation of epigenetic regulation, particularly through histone modifications, provides an additional strategy to increase gene knock‐in efficiency. For example, histone deacetylase inhibitors (HDACis), either alone or in combination with NHEJ inhibitors, significantly increase HDR efficiency [31]. Notably, histone modifications at DSBs undergo highly dynamic and coordinated regulation rather than isolated changes. With the development of epigenome editing technologies, locus‐specific manipulation of chromatin states has become feasible, suggesting the possibility that targeted modulation of histone modifications could be harnessed to improve precise gene editing. However, strategies that increase CRISPR–Cas9‐mediated gene knock‐in efficiency through the regulation of histone modifications remain underexplored.

Current strategies for improving CRISPR–Cas9‐mediated knock‐in efficiency have largely focused on inhibiting NHEJ factors, modulating the cell cycle, or optimizing the CRISPR‐Cas9 system. While these approaches can be effective, their efficiencies are often highly variable across different genomic loci. Previous studies have demonstrated that chromatin modification is closely linked to DNA repair [36, 37]. Here, we propose that modulating the chromatin environment represents a largely underexplored yet powerful strategy. We synthesize these emerging epigenetic insights to build a conceptual framework centred on histone modifications at the DSB sites. By elucidating how specific histone marks at DSBs influence HDR and NHEJ dynamics, we ultimately propose prospective epigenetic strategies for improving the efficiency of precise gene editing.

## 2 Current Strategies for Improving CRISPR–Cas9‐Mediated Knock‐In Efficiency

The CRISPR–Cas9 system functions through the formation of a dual‐RNA complex composed of CRISPR RNA (crRNA) and trans‐activating crRNA (tracrRNA), which guides the Cas9 endonuclease to specific genomic loci via base pairing with the target DNA sequence. Upon recognition of a protospacer adjacent motif (PAM, whose DNA sequence is NGG), Cas9 induces site‐specific DSBs through its HNH and RuvC nuclease domains [38, 39]. DSBs generated by CRISPR–Cas9 are subsequently repaired through complex intracellular DNA repair pathways, primarily error‐prone end‐joining mechanisms or error‐free HDR. NHEJ and MMEJ operate throughout the cell cycle and frequently introduce insertions or deletions (indels), whereas HDR enables precise sequence replacement or insertion via homologous DNA templates [40, 41, 42].

Despite its precision, HDR is intrinsically inefficient. It is restricted to the S/G2 phases of the cell cycle, requires donor templates, and proceeds through a multistep and time‐consuming process that can last several hours [43, 44]. Consequently, CRISPR–Cas9‐mediated HDR efficiencies typically range from 0.5% to 20%, substantially lower than those of NHEJ‐mediated editing, which often exceeds 20%–70% in mammalian systems [42, 45]. Because HDR‐mediated gene knock‐in offers superior precision and safety compared with other gene integration methods, increasing studies have focused on improving CRISPR–Cas9‐mediated knock‐in efficiency. Based on current insights into DNA repair pathway choice and the molecular mechanisms governing NHEJ and HDR, these strategies can be broadly classified into three major methods.

### 2.1 Optimization of CRISPR–Cas9 Systems to Increase Gene Knock‐In Efficiency

Modification of CRISPR–Cas9 system components has been explored to increase knock‐in efficiency. These approaches include optimization of the sgRNA structure, engineering of Cas9 variants, and modification of donor DNA templates. Modified sgRNAs can improve the stability of the Cas9–RNA–DNA complex, increase editing efficiency, and reduce off‐target effects. Cas9 variants with altered DNA‐binding or cleavage properties have been shown to reduce off‐target activity [46]. However, multiple studies have reported that increased nuclease specificity or activity alone often has a minimal effect on HDR‐mediated knock‐in efficiency [33, 47]. With respect to donor template design, single‐stranded oligodeoxynucleotides (ssODNs) are frequently used to improve HDR efficiency and reduce unwanted integration at cleavage sites [16, 29, 48]. The incorporation of Cas9‐targeting sequences (CTSs) within the ssODN homology arms has been reported to increase the knock‐in efficiency by more than twofold [17, 31]. In addition, negatively charged anionic polymers such as poly‐l‐glutamic acid (PGA) have been employed to stabilize ribonucleoprotein (RNP)‐donor DNA complexes by preventing aggregation and precipitation, thereby maintaining cell viability while increasing knock‐in efficiency [49]. However, conflicting reports indicate that the PGA does not consistently enhance HDR outcomes [30], highlighting the need for further methodological optimization. Moreover, large‐scale ssDNA production remains costly compared with that of double‐stranded DNA, requiring technological advances in ssDNA synthesis and cost reduction (Figure 1a).

**FIGURE 1:**
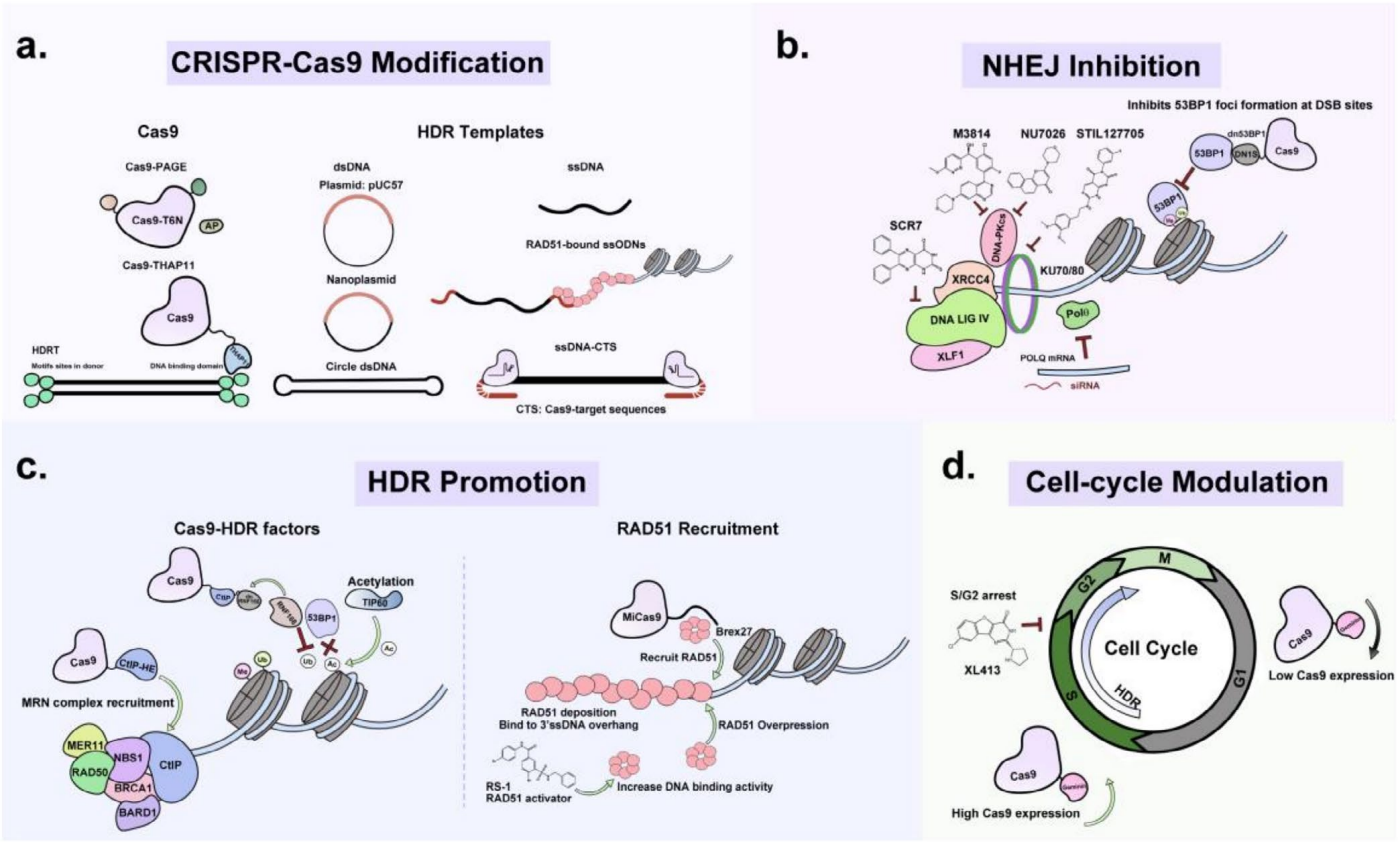
Current strategies for enhancing CRISPR–Cas9‐mediated HDR. (a) Cas9 engineering and HDR template delivery strategies. Engineered Cas9 fusion systems and optimized donor template designs improve precise knock‐in efficiency. Cas9 fusions include peptide‐ or protein‐assisted systems such as Cas9–PAGE (Peptide‐Assisted Genome Editing CRISPR–Cas system; T6N fused N‐terminally to HIV TAT and 4× Myc nuclear localization signals (NLSs), and C‐terminally to 2× SV40 NLSs and GFP; AP, assistant peptide) and Cas9–THAP11 (THAP domain‐containing protein 11 DNA‐binding domain). HDR donor templates include double‐stranded DNA (dsDNA; e.g., pUC57 plasmid, nanoplasmid, and circular dsDNA) and single‐stranded DNA (ssDNA; e.g., long ssDNA, ssODNs, RAD51‐bound ssODNs, and ssDNA containing Cas9 target sequences (ssDNA‐CTS)). (b) Inhibition of the NHEJ pathway. Suppression of NHEJ shifts the choices of the DNA repair pathway toward HDR. Strategies include small‐molecule inhibitors targeting key NHEJ factors, such as the DNA ligase IV inhibitor SCR7; the Ku70/80 inhibitor STL127705; and the DNA‐PKcs inhibitors M3814 and NU7026. Cas9 fusion with dominant‐negative 53BP1 (Cas9‐dn53BP1), which inhibits 53BP1 foci formation at DSBs, and inhibition of DNA polymerase theta (Pol θ/POLQ), for example via POLQ siRNA. (c) Direct enhancement of HDR components includes Cas9 fusion with HDR factors such as CtIP (recruiting the MRE11–RAD50–NBS1 (MRN) complex), HE (the N‐terminal domain of CtIP), CtIP‐dnRNF168 (inhibition of H2AK15 ubiquitination impairs 53BP1 recruitment to DSBs and promotes TIP60‐mediated histone acetylation), and Brex27 (BRCA2 exon 27). Additional strategies stimulate RAD51 activity through small‐molecule activators (e.g., RS‐1) or RAD51 overexpression. (d) Restricting Cas9 activity to S/G2 phases, when HDR is most active, improves the precise knock‐in efficiency. Approaches include Cas9 fusion with S/G2 phase‐specific proteins (e.g., Geminin) and pharmacological induction of S/G2 arrest (e.g., the CDC7 inhibitor XL413).

### 2.2 Modulation of DNA Repair Pathways to Increase Gene Knock‐In Efficiency

Modulation of DNA repair pathway activity represents another major strategy to promote HDR. Inhibition of NHEJ‐associated factors, including Ku70/80, DNA‐PKcs, DNA ligase IV and DNA polymerase θ, has been shown to bias repair outcomes toward homologous recombination [16, 21, 50, 51, 52]. Small‐molecule inhibitors of DNA‐PKcs, such as NU7026 and M3814, can increase HDR efficiency. However, accumulating evidence indicates that global inhibition of the NHEJ pathway can compromise genome integrity. For example, treatment with AZD7648, a potent DNA‐PKcs inhibitor, while improving gene knock‐in efficiency, induces frequent kilobase‐ to megabase‐ scale deletions, chromosomal arm losses, and translocations. Such large‐scale chromosomal alterations are difficult to detect via usual genome editing assays [23]. Therefore, when AZD7648‐like inhibitors are used to enhance HDR, a comprehensive evaluation of diverse editing outcomes. In parallel, increasing the expression or localized recruitment of HDR‐associated factors can also facilitate HDR. Fusion of Cas9 with HDR‐related proteins or functional domains, including RAD51, RAD52, CtIP, mSA, or DN1S, has been shown to significantly increase the HDR frequency [53, 54, 55, 56, 57, 58]. Similarly, Cas9 fused to short peptides that selectively recruit RAD51 (e.g., MiCas9) directly promotes HDR at DSB sites [33]. Nevertheless, identifying a fusion structure that preserves Cas9 nuclease activity while avoiding unintended perturbation of endogenous DNA repair processes remains technically challenging (Figure 1b,c).

### 2.3 Modulation of Cell Cycle to Increase Gene Knock‐In Efficiency

The regulation of the cell cycle represents another commonly used strategy to improve gene knock‐in efficiency. Pharmacological prolongation of the S/G2 phase can favour HDR‐mediated repair and increase precise integration efficiency [24]. Alternatively, the fusion of Cas9 to the N‐terminal region of human Geminin renders this nuclease a substrate of the APC/Cdh1 E3 ubiquitin ligase complex, resulting in low Cas9 expression during G1 and preferential activity during the S/G2/M phases. Compared with wild‐type Cas9, this approach has been reported to improve HDR efficiency by up to 87% [59, 60]. Despite these advantages, cell cycle‐restricted Cas9 strategies are limited by narrow editing windows, cell‐type dependence, potential off‐target effects, technical complexity, and possible interference with endogenous DNA repair pathways. Further optimization is needed to broaden their applicability and robustness (Figure 1d).

## 3 Chromatin Structure and DNA Repair Pathway Choice

In recent years, advances in epigenetic technologies have revealed a close association between chromatin state and HDR‐mediated DNA repair. Accumulating evidence indicates that histone modifications and their dynamic regulation play critical roles in regulating repair pathway choice following DSBs. By modulating nucleosome stability, DNA accessibility, and higher‐order chromatin architecture, these modifications influence the recruitment, assembly, and coordination of the repair machinery. As a result, the chromatin landscape surrounding DSBs emerges as a critical determinant of repair outcomes and is capable of promoting or constraining HDR factors [36]. These insights provide a conceptual framework for increasing gene knock‐in efficiency through targeted chromatin modulation.

### 3.1 Histone Posttranslational Modifications Coordinate DSB Repair Pathway Choice

In eukaryotic cells, genomic DNA is packaged into chromatin, a nucleoprotein complex that both compacts the genome and regulates DNA‐dependent processes. The nucleosome, which is composed of H2A, H2B, H3, and H4 wrapped by ~147 bp of DNA and stabilized by histone H1, provides structural organization while influencing DNA damage recognition and repair [61]. Chromatin is organized into euchromatin and heterochromatin, which differ in compaction, transcriptional activity, and epigenetic features, thereby shaping cellular responses to DSBs. Rather than acting as a structural framework, chromatin dynamically remodels following DSB induction through alterations in nucleosome positioning, histone variants, and posttranslational modifications (PTMs), ultimately regulating DNA accessibility, repair factor recruitment, and repair pathway choice [36].

DSBs in euchromatic regions are typically repaired more rapidly and can engage either NHEJ or HDR, whereas heterochromatic breaks exhibit delayed repair and chromatin‐dependent pathway biases, with H3K27me3‐enriched regions favouring MMEJ and H3K9me3‐marked domains showing increased HDR [37]. Genome‐wide studies further revealed that chromatin remodelling extends over kilobase regions around DSBs, indicating spatially organized epigenetic responses [36]. In addition to histone modifications, DNA methylation also participates in DSB responses. Following damage, DNMT1 is recruited near DSBs, leading to 5mC deposition, which can be converted to 5hmC by TET enzymes. 5hmC colocalizes with γH2AX and 53BP1 and may support genome stability [62, 63] However, direct evidence linking DNA methylation to repair pathway choice remains limited. Compared with DNA methylation, histone modification has a more transient impact on DSB repair, as they highly dynamic and reversible. Because they can be rapidly deposited or removed after damage, histone modifications promptly alter chromatin structure and promote the recruitment of repair factors, thereby influencing repair pathway choice.

Histone PTMs, including methylation, acetylation, phosphorylation, ubiquitination, and SUMOylation, serve as molecular signals that integrate DNA damage sensing with chromatin remodelling and repair factor recruitment. Upon DSB induction, a cascade of histone modifications is rapidly established at and around DSBs, creating a dynamic chromatin state [64]. First, they modulate chromatin accessibility by altering nucleosome stability and higher‐order chromatin folding. Second, they provide binding platforms for epigenetic readers and DNA repair proteins. Third, they influence the stability, retention, and activity of repair complexes, thereby biasing repair pathway choice. Importantly, both preexisting histone marks and damage‐induced PTM remodelling contribute to repair outcomes, reflecting the context‐dependent nature of DNA repair regulation. Epigenetic ‘writers’, ‘erasers’, and ‘readers’ play critical roles in this process. Histone acetyltransferases (HATs) and deacetylases (HDACs) regulate chromatin relaxation near DSBs, facilitating DNA end resection and increasing the accessibility of repair factors. Acetylated histones also recruit nucleosome remodelling complexes, further promoting DSB processing [36, 65, 66, 67]. In parallel, histone methyltransferases and demethylases govern the recruitment and stability of key repair proteins, including CtIP, 53BP1, and BRCA1, thereby influencing the balance between the NHEJ and HDR pathways [68, 69, 70, 71]. Ubiquitination and SUMOylation cascades reinforce these processes by coordinating chromatin reorganization with DNA damage checkpoint activation [72]. Collectively, these epigenetic mechanisms generate a dynamic chromatin landscape that actively directs the choice of DSB repair pathway.

### 3.2 Histone Modifications Associated With Enhanced HDR

Accumulating evidence suggests that specific histone modifications create a chromatin environment permissive for HDR. Trimethylation of histone H3 lysine 36 (H3K36me3) is a hallmark of actively transcribed euchromatin and plays a pivotal role in promoting HDR. Following DSB formation, H3K36me3 is recognized by the PWWP domain‐containing protein lens epithelium‐derived growth factor (LEDGF), which recruits CtIP to damage sites, thereby facilitating DNA end resection. This process enables the subsequent binding of replication protein A (RPA) and RAD51, driving homologous recombination [68]. Notably, H3K36me3 levels remain largely unchanged following DSB induction [73], indicating that this mark primarily acts as a preexisting epigenetic signal that licenses HDR in euchromatic regions (Figure 2a).

**FIGURE 2:**
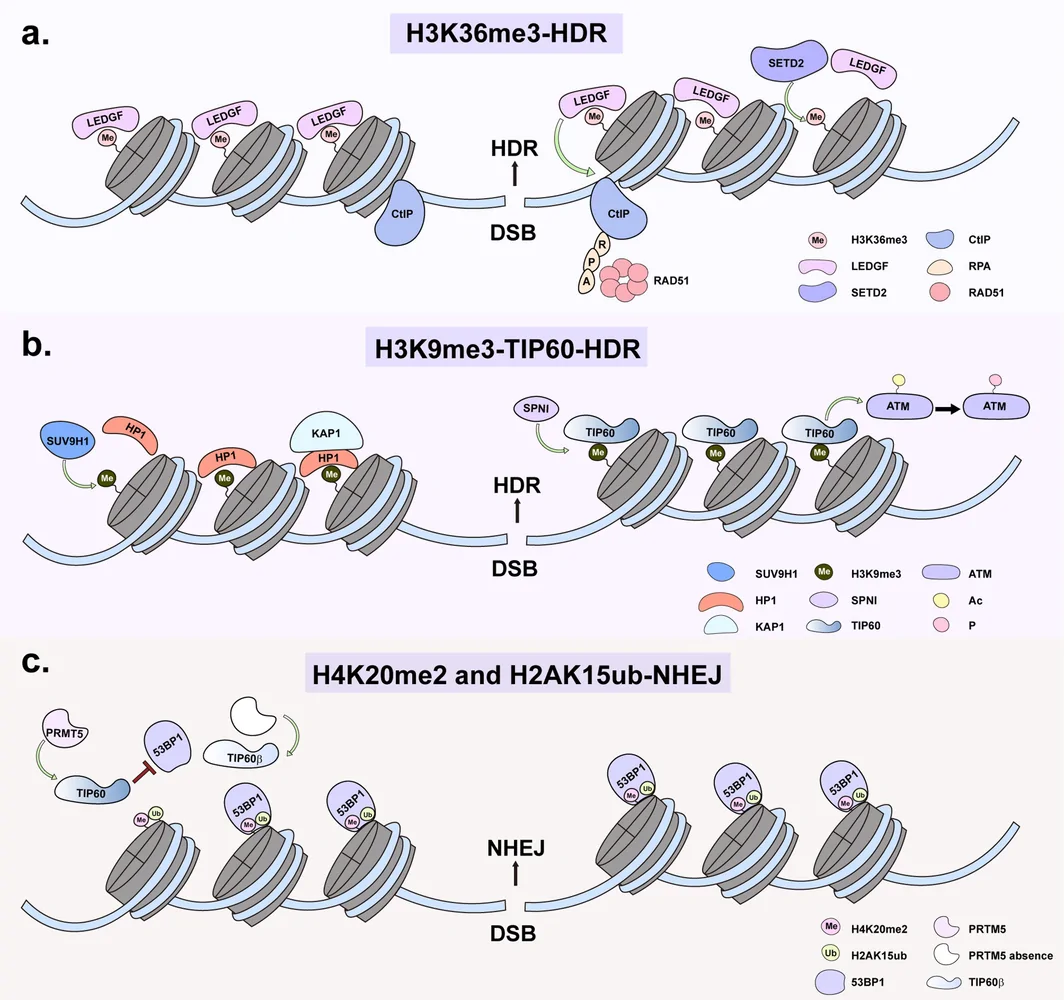
Histone modifications orchestrate DSB repair pathway choice. (a) H3K36me3 promotes HDR at DSBs. Following DSB induction, SETD2‐mediated H3K36me3 is recognized by LEDGF, which recruits CtIP to DSBs to initiate DNA end resection. RPA binds and stabilizes the resulting ssDNA and is subsequently replaced by RAD51 to promote HDR. (b) The H3K9me3‐TIP60 axis promotes HDR. SUV39H1 mediates H3K9me3 deposition near DSBs. H3K9me3 recruits the HP1‐KAP1 complex to stabilize local chromatin structure and facilitates TIP60 recruitment, potentially through SPIN1. TIP60‐mediated ATM acetylation promotes ATM phosphorylation and activation of the DNA damage response, thereby favouring HDR. (c) H4K20me2 and H2AK15ub promote NHEJ. H4K20me2 and H2AK15ub at DSBs recruit 53BP1, restrict DNA end resection, and promote NHEJ. PRMT5 loss increases TIP60β production, enhancing 53BP1 accumulation at DSBs and shifting repair pathway choice toward NHEJ.

H3K9 trimethylation (H3K9me3) is a classical hallmark of transcriptionally repressive heterochromatin and has long been associated with reduced DNA accessibility. Paradoxically, accumulating evidence indicates that H3K9me3 plays an essential and active role in promoting HDR following DSBs, particularly through activation of the ATM‐TIP60 signalling axis. Upon DSB induction, the histone methyltransferase SUV39H1 is rapidly recruited to damage sites, where it catalyses local H3K9me3 deposition. This initial nucleation event promotes the assembly of KAP1/HP1/SUV39H1 complexes and facilitates the spread of H3K9me3 along chromatin regions flanking the break [74]. Such damage‐induced heterochromatinization serves as a signalling platform required for efficient DNA damage response activation.

A key mechanistic connection between H3K9me3 and HDR is mediated by TIP60, a histone acetyltransferase. H3K9me3 enhances the interaction between TIP60 and chromatin, leading to TIP60‐dependent acetylation of histone H4 and subsequent activation of ATM kinase. Activated ATM phosphorylates downstream substrates, including γH2AX and KAP1, thereby promoting chromatin relaxation at later stages and enabling end resection and HDR factor recruitment [75]. Loss of H3K9me3 impairs ATM activation, disrupts H4 acetylation dynamics, and results in defective DSB repair and genomic instability [74], reflecting the functional necessity of this modification in HDR. Recent studies further identified Spindlin1 (SPIN1) as an epigenetic reader that binds H3K9me3 through its Tudor‐like domains and facilitates TIP60 activation at DSBs. SPIN1‐dependent reinforcement of H3K9me3‐TIP60 interactions promotes efficient ATM signalling and HDR, whereas SPIN1 deficiency leads to delayed repair kinetics and increased genomic instability [76]. Together, these findings reveal that transient, damage‐induced heterochromatin formation represents an essential intermediate state in HDR rather than an obstacle to repair (Figure 2b).

### 3.3 Histone Modifications Associated With Enhanced 53BP1‐Mediated NHEJ

The balance between NHEJ and HDR is critically regulated by the recruitment of 53BP1, a key factor of end‐joining repair. 53BP1 localization to DSBs relies on dual recognition of histone H4K20 dimethylation (H4K20me2) and the ubiquitination of H2AK15 (H2AK15ub), forming a stable chromatin platform that favours NHEJ and suppresses DNA end resection [77, 78, 79]. The TIP60 acetyltransferase complex counteracts 53BP1‐mediated NHEJ through multiple mechanisms. TIP60‐dependent acetylation of histone H4 weakens 53BP1 binding to H4K20me2 and modulates H2AK15 ubiquitination, thereby creating a chromatin environment permissive for BRCA1–BARD1 recruitment and HDR initiation [80]. Importantly, TIP60 function is regulated by PRMT5. In the absence of PRMT5, a splice variant of TIP60 (TIP60β) is generated that fails to effectively compete with 53BP1 for chromatin binding, resulting in a shift toward NHEJ‐mediated repair [81]. Collectively, these observations highlight that preexisting histone modifications, damage‐induced chromatin remodelling, and dynamic regulation of histone‐modifying enzymes form an integrated epigenetic network that governs DSB repair pathway choice (Figure 2c).

## 4 Epigenetic Modulation Strategies to Increase Knock‐In Efficiency

The emergence of gene‐editing technologies has greatly expanded the precision and range of epigenetic research. In particular, CRISPR‐based epigenome‐editing tools, in which nuclease‐dead Cas9 (dCas9) is fused to epigenetic modifiers, enable locus‐specific manipulation of chromatin states without altering the underlying DNA sequence [82]. For example, the dCas9‐DNMT3A and dCas9‐TET1 systems have been widely used to explore the roles of DNA methylation and demethylation in transcriptional repression and activation, respectively [83, 84, 85]. Similarly, the fusion of dCas9 with histone‐modifying enzymes, such as LSD1 or the histone acetyltransferase p300, allows the targeted removal or deposition of histone marks at promoters and enhancers, providing powerful tools for dissecting chromatin‐dependent gene regulation [86, 87] (Figure 3a,b).

**FIGURE 3:**
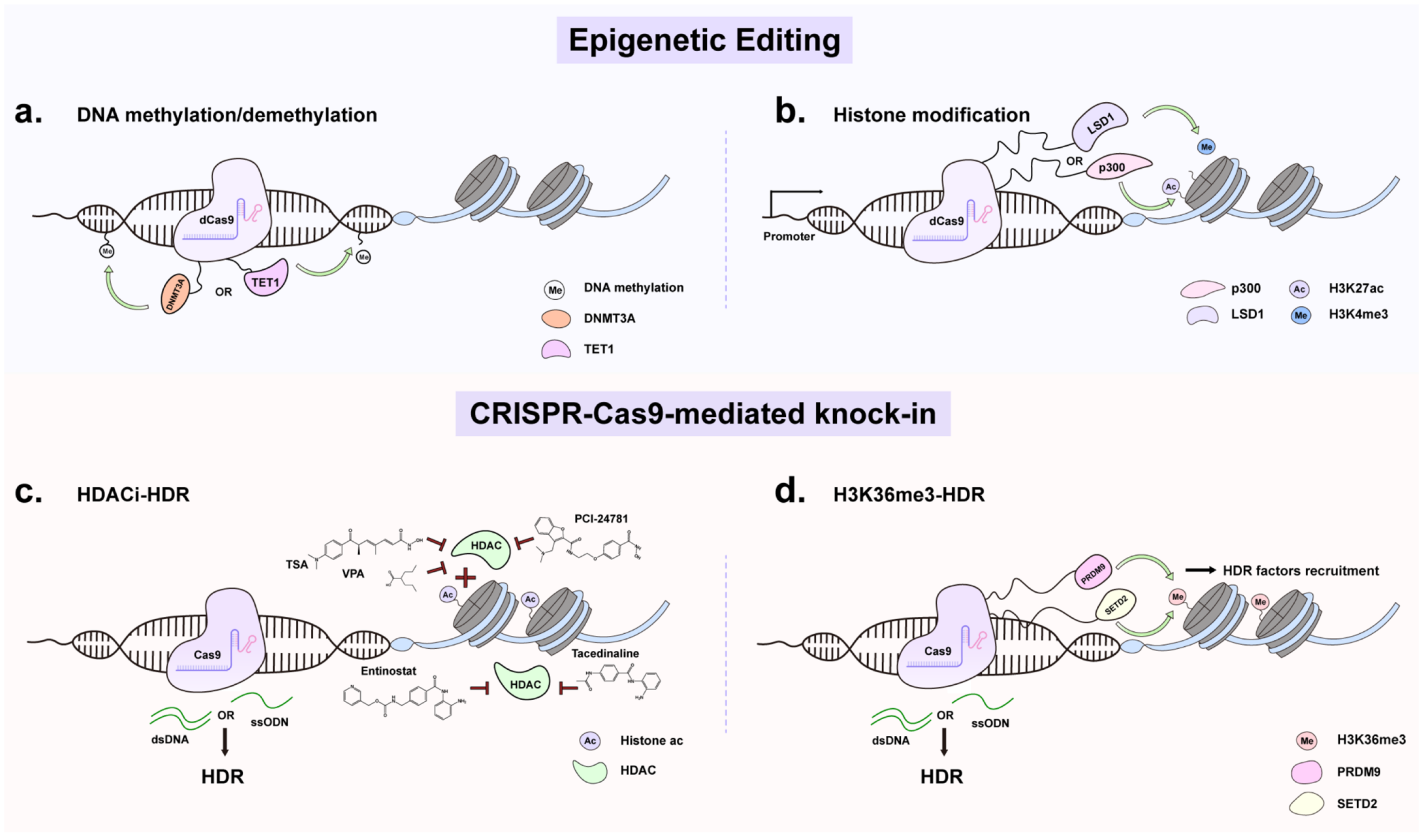
CRISPR‐based epigenetic engineering and epigenetic modulation of CRISPR–Cas9‐mediated knock‐in. (a, b) CRISPR‐based epigenetic engineering. Catalytically inactive Cas9 (dCas9) fused to epigenetic effector domains enables locus‐specific modulation of chromatin states without inducing DNA DSBs. (a) Fusion of dCas9 to DNMT3A or TET1 induces targeted DNA methylation or demethylation, leading to transcriptional repression or activation, respectively. (b) Similarly, coupling dCas9 to histone‐modifying enzymes including histone acetyltransferases (e.g., p300) or histone demethylases (e.g., LSD1) enables precise manipulation of histone acetylation or methylation states. These programmable systems allow targeted activation or silencing of gene expression and precise remodelling of the epigenetic landscape. (c, d) Chromatin context, modulated by histone modifications, critically influences DSB repair pathway choice and gene knock‐in efficiency. Strategies include (c), pharmacological inhibition of histone deacetylases (e.g., TSA, VPA, PCI‐24781, entinostat, and tacedinaline) to increase accessibility. (d) Fusion of Cas9 to PRDM9 or SETD2 to promote locus‐specific H3K36me3 deposition, facilitating the recruitment of HDR‐associated repair factors to DSBs.

In addition to their utility in epigenetics, CRISPR–Cas9‐based platforms have revealed tight coupling between local chromatin states and DNA damage repair pathway choice. Because Cas9‐induced DSBs are generated and repaired within the endogenous chromatin environment, the epigenetic landscape surrounding the break site critically influences the recruitment and activity of DNA repair factors. On the basis of the epigenetic mechanisms governing DSB repair pathway choice, multiple strategies may be employed to bias Cas9‐induced DNA repair toward HDR. These approaches can be broadly divided into three types: (i) modulation of chromatin accessibility and histone modification states, (ii) targeted recruitment of epigenetic regulators to Cas9 break sites, and (iii) attenuation of chromatin‐dependent barriers to HDR, particularly 53BP1‐mediated end‐joining. Together, these strategies aim to transiently create a chromatin environment that favours DNA end resection and HDR while minimizing genome‐wide perturbations.

### 4.1 Regulating Chromatin Accessibility to Improve Knock‐In Efficiency

Chromatin accessibility reflects the ability of nuclear macromolecules to physically access chromatinized DNA and is primarily determined by nucleosome occupancy, histone modifications, and higher‐order chromatin organization [88]. In interphase cells, most genomic regions are packaged into compact heterochromatin, whereas only a limited fraction exist as transcriptionally permissive euchromatin. This dynamic accessibility is essential for cellular processes, including transcription, DNA replication, and DNA damage repair, and is tightly coupled to epigenetic modification states. Because CRISPR–Cas9‐induced DSBs are generated and repaired within native chromatin contexts, local chromatin accessibility critically influences Cas9 binding and cleavage efficiency, as well as downstream DNA repair pathway choice. Accessible chromatin facilitates Cas9 recruitment and cleavage, enhances DNA end resection, and promotes the loading of HDR factors such as RAD51, thereby biasing repair toward HDR, whereas rapid KU binding under limited resection conditions can favour NHEJ [89, 90, 91].

HDAC inhibitors (HDACi) are a widely used strategy for transiently increasing chromatin accessibility. They promote histone hyperacetylation, chromatin decompaction, and increased accessibility at Cas9 target sites. High‐throughput screening of epigenetic inhibitors across multiple chromatin contexts revealed that HDACis partially enhance Cas9 editing outcomes [92], although their effects depend strongly on local chromatin states. For example, trichostatin A (TSA) preferentially enhanced Cas9 editing efficiency at loci with low basal H3K27ac levels, while exerting weaker effects at already acetylated regions, highlighting the site‐dependent nature of HDACi action. In primary fibroblasts, TSA and PCI‐24781 increased knock‐in efficiency by approximately twofold at the GAPDH locus and 1.6–2‐fold at the *ACTB* locus, with variability across genomic sites. These effects likely reflect a combination of chromatin decompaction, altered acetylation of NHEJ factors such as KU70/80, and shifts in cell‐cycle phase distribution that favour HDR [93, 94]. Notably, when TSA was combined with inhibitors targeting complementary pathways, the knock‐in efficiency at the *CD5* locus in primary T cells was further increased, reaching a 134% increase compared with that of the control conditions [31]. Valproic acid (VPA), another HDAC inhibitor, increases the acetylation of H3K9, H3K27, and H4K16 by suppressing HDAC2 activity, thereby loosening the nucleosome structure at transcriptionally inactive loci. At the *AAVS1* site, VPA treatment increased HDR efficiency by approximately twofold, and even enabled homozygous editing at the otherwise refractory *HNF4α* locus [95]. Moreover, combined treatment with HDAC inhibitors such as VPA, sodium butyrate (NaB), TSA, and suberoylanilide hydroxamic acid (SAHA; also known as vorinostat) further increased Cas9 and sgRNA expression levels, improving knock‐in efficiency at both open and closed chromatin regions, although the NHEJ frequency was also elevated [96]. Other HDAC inhibitors, including entinostat and tacedinaline, similarly increase HDR efficiency while reducing NHEJ frequency, although their precise molecular mechanisms remain incompletely understood [97]. However, because HDACis induce global histone hyperacetylation rather than locus‐specific remodelling, they may increase off‐target editing and broader epigenomic perturbations, highlighting the need for more precise, locus‐restricted epigenetic modulation strategies to increase HDR without globally disrupting the chromatin architecture (Figure 3c and Table 1).

**TABLE 1:** Histone modification‐based strategies for CRISPR–Cas9 knock‐in.

Strategy	Treatment	Mechanism	Edited locus	Delivery of Cas9	Donor type	Cell	Effect	Limitations	References
HDAC Inhibition	Small molecule: VPA	Increases H3K9, H3K27, and H4K16 acetylation, thereby promoting chromatin relaxation.	*AAVS1/HNF4α/FOXA2*	Plasmid	Plasmid	iPSC/ES	HDR↑	Potential global chromatin decondensation and off‐target effects; limited pathway specificity due to concurrent NHEJ and HDR activation; primarily applicable to plasmid‐based systems; mechanisms remain incompletely defined.	[95]
	Small molecule: TSA	Increases acetylation levels of H3K9 and H3K27, promoting chromatin accessibility.	*ACTL6A.5*	Cas9‐expressing cell line		HepG2	HDR↑		[94]
	Small molecules: TSA PCI‐24781	Increases chromatin accessibility, reduces binding of NHEJ factors to DNA, and induces G2/M cell cycle arrest.	*GAPDH/ACTB*	Plasmid/RNP	dSDNA/ssODN	Pig embryo/Pig foetal fibroblasts	HDR↑ c‐NHEJ↓		[93]
	Small molecules: NaB/TSA/SAHA/VPA	Increases chromatin accessibility and elevates Cas9 and sgRNA expression levels.	Multiple loci	Plasmid	Plasmid	iPSC	HDR↑ NHEJ↑		[96]
	Small molecules: TSA/M3814/XL413	Increases chromatin accessibility, inhibits NHEJ pathway and induces S/G2 phase cell cycle arrest	Multiple loci	RNP	ssODN	Primary T	HDR↑		[31]
	Small molecules: Entinostat/Tacedinaline	Upregulates Cas9 and sgRNA expression levels.	Multiple loci	Cas9‐expressing cell lines	Plasmid	HEK293T/Mouse retina	HDR↑ NHEJ↓		[97]
Increasing H3K36me3 Levels	Cas9 fusion: Cas9‐PRDM9/SETD2	Site‐specific deposition of H3K36me3 and H3K4me3	Multiple loci	Plasmid	Plasmid/ssODN	HEK293T/HeLa/U2OS/IMR90	HDR↑	Limited applicability at loci with high endogenous H3K36me3 levels.	[73]

Given the limitations of global HDAC inhibition, targeted enhancement of histone acetylation at Cas9 cleavage sites has been explored via CRISPR‐based epigenome editing. dCas9 fused to transcriptional activation domains (e.g., VP64) or histone acetyltransferases (e.g., p300) has been successfully used to activate gene expression [87, 98]. However, when these domains were fused to nuclease‐active Cas9, unexpected inhibitory effects on both indel formation and HDR were observed. In particular, Cas9‐p300 fusions markedly reduce editing efficiency, likely due to steric hindrance, reduced RNP mobility, or compromised protein stability [96]. Future optimization strategies may include the use of truncated or modular acetylation domains, sgRNA‐associated peptide recruitment systems, or non‐plasmid delivery methods (e.g., mRNA or RNP delivery) to preserve Cas9 activity while achieving transient, localized chromatin opening. However, these negative results also reveal that it may not be due to the failure of the fusion protein strategy. The phenomenon that Cas9‐p300 fails to increase the knock‐in efficiency indicates that the relationship between chromatin accessibility and DNA repair is both non‐linear and highly complex. Inappropriate chromatin opening may also be detrimental to HDR. On the contrary, it may cause a series of negative effects, such as the increase in chromatin openness promoting the activation of the NHEJ pathway and thereby suppressing HDR. Therefore, while enhancing chromatin accessibility remains a promising strategy for increasing gene knock‐in efficiency. It is necessary to carefully evaluate the chromatin modification status before and after editing and conduct an objective discussion of the negative results to build a more predictive and effective framework.

### 4.2 Regulation of Histone Modifications to Bias DNA Repair Toward HDR

While modulation of chromatin accessibility and histone acetylation provides an effective means to improve CRISPR–Cas9 knock‐in efficiency, accessibility is insufficient to fully explain repair pathway choice across different genomic contexts. Increasing evidence indicates that specific histone modification signatures at DSB sites actively instruct the balance between HDR and NHEJ, independent of global chromatin accessibility. Accordingly, recent strategies have focused on direct manipulation of histone methylation and ubiquitination marks to selectively promote HDR while suppressing NHEJ, offering a more precise approach to improve gene knock‐in efficiency.

H3K36me3 is a well‐established chromatin mark associated with transcriptional elongation and homologous recombination. Genome‐wide analyses of AsiSI‐induced DSBs have demonstrated that breaks occurring within H3K36me3‐enriched regions are preferentially repaired through HDR [36], highlighting its role in repair pathway choice. To exploit this property for gene editing, PRDM9 was fused to Cas9, enabling targeted deposition of H3K36me3 and H3K4me3 at cleavage sites that normally lack these modifications. PRDM9‐Cas9 significantly increased HDR efficiency (up to threefold) and improved the HDR‐to‐indel ratio by as much as fivefold without elevating off‐target editing. Notably, the enhancement was minimal at loci already enriched for H3K36me3, suggesting that additional deposition of this mark is beneficial only in chromatin contexts where it is limiting. In addition to its catalytic activity, PRDM9 may also recruit the chromatin remodeller HELLS, further facilitating DNA end resection and HDR. Similarly, the fusion of SETD2, the primary H3K36me3 methyltransferase in mammalian cells, to Cas9 enhances HDR efficiency and promotes DNA end resection by recruiting CtIP via H3K36me3‐dependent interactions. However, not all H3K36 methyltransferases exert equivalent effects. For example, SETMAR‐Cas9 failed to promote HDR, likely reflecting its relatively weak methyltransferase activity. These observations demonstrate the importance of enzyme specificity and catalytic potency when designing histone‐modifying Cas9 fusions [73] (Figure 3d and Table 1).

## 5 Potential Conceptual Strategies for Regulating Histone Modifications to Enhance Gene Knock‐In Efficiency

Although CRISPR‐based tools have been widely applied to study epigenetic mechanisms, their potential to utilize epigenetic modifications or associated enzymes to actively improve gene knock‐in remains largely unexplored. As discussed in the previous section, the chromatin context and epigenetic landscape critically influence DNA repair pathways, resulting in pronounced locus‐dependent variability in knock‐in efficiency. These observations suggest that the rational modulation of histone modification enzymes or epigenetically coupled DNA repair factors may represent a promising and potentially powerful strategy for improving knock‐in efficiency. In this section, we explore conceptual regulatory strategies based on histone modifications that may enhance precise gene integration, elucidating their underlying regulatory mechanisms and potential challenges.

### 5.1 H3K36me3‐Based Strategies to Enhance HDR‐Mediated Knock‐In

In addition to directly depositing H3K36me3 at target loci via PRDM9‐ or dCas9‐SETD2 fusion systems, a hypothetical future direction for increasing H3K36me3 levels by inhibiting H3K36me3 demethylases. The KDM4 family, particularly KDM4A and KDM4B, represents a potential target strategy, as they are responsible for H3K36me3 and H3K9me3 demethylation [99]. Functional studies provide evidence for this predictive strategy. KDM4B is closely linked to HDR, as its depletion impairs TIP60 and RAD51 foci formation, whereas KDM4A appears to have minimal direct effects on HDR but is important for cell cycle progression and replication dynamics. KDM4A overexpression leads to faster S‐phase progression, accelerated DNA replication and transcription, and decreased H3K9me3 and H3K36me3 levels [100, 101]. One could hypothesize that short‐term suppression of KDM4A activity might create a chromatin environment that favours HDR and potentially enhance gene knock‐in efficiency. However, global inhibition of KDM4A would likely induce widespread chromatin alterations and unintended transcriptional effects due to the family's broad substrate specificity and non‐catalytic domain functions. To mitigate the potential risks, hypothetical site‐restricted approaches could be considered. For example, Cas9‐tethered inhibitory peptides targeting the JmjC catalytic domain of KDM4A could locally attenuate demethylase activity at DSB sites. Alternatively, transient siRNA‐mediated knockdown of KDM4A prior to or during editing might offer temporal control. If proven effective, such strategies would aim to reduce off‐target chromatin remodelling while preserving the HDR‐promoting benefits of elevated H3K36me3 (Figure 4a).

**FIGURE 4:**
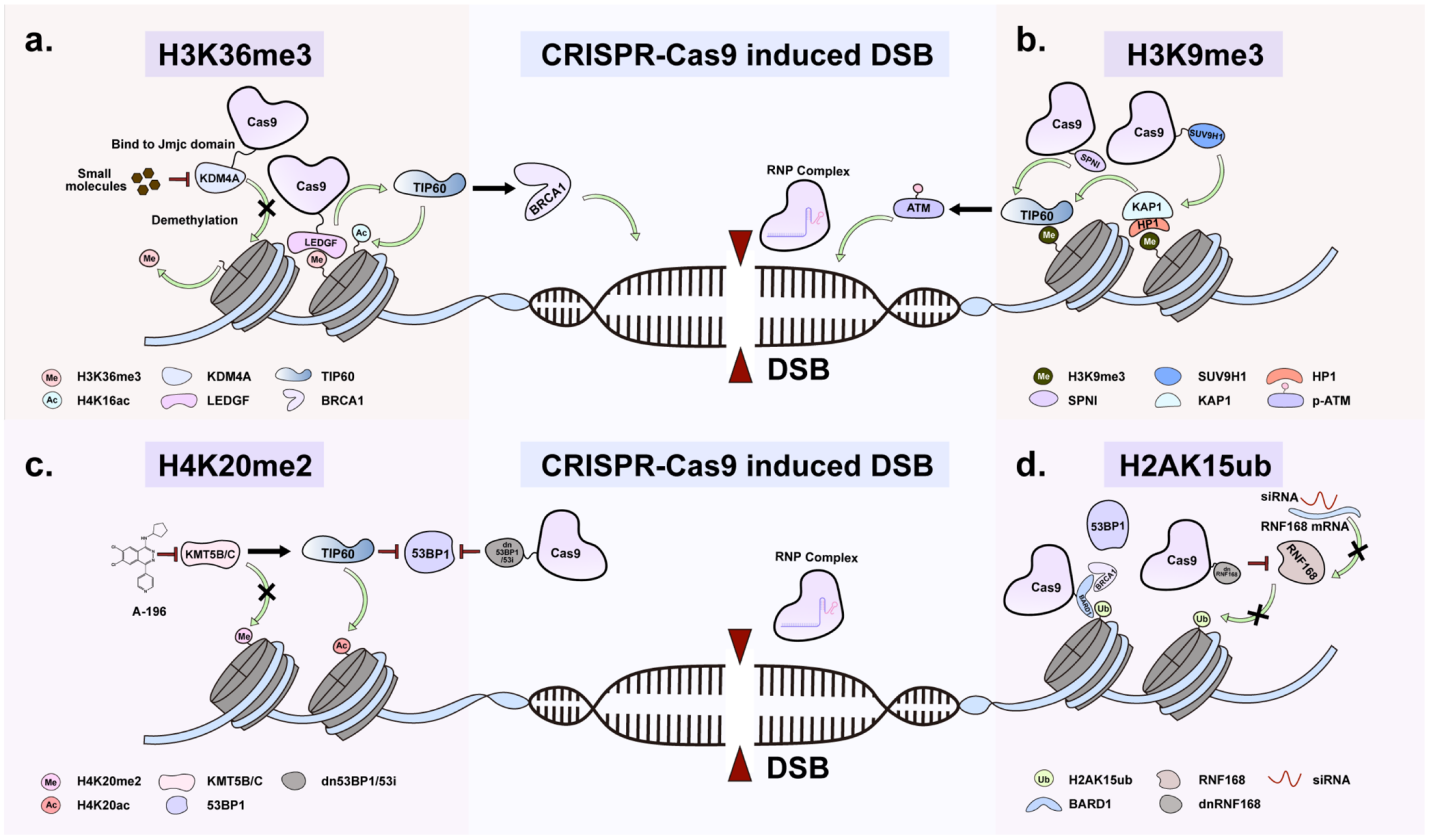
Proposed epigenetic modulation strategies to improve CRISPR–Cas9‐mediated HDR. (a, b) Targeted modulation of histone modifications to bias CRISPR‐induced breaks toward HDR. (a) Hypothetical strategies to increase H3K36me3 at DSBs to favour HDR. One proposed approach is the transient inhibition of the H3K36 demethylase KDM4A via small‐molecule inhibitors. It is hypothesized that this could be achieved more locally through Cas9‐mediated delivery of inhibitory peptides targeting the KDM4A Jumonji C (JmjC) domain. A related hypothetical strategy involves a Cas9‐LEDGF fusion, which is theorized to enhance recognition of H3K36me3 at DSBs, thereby promoting TIP60‐mediated H4K16ac and facilitating the recruitment of HDR factors such as BRCA1. (b) Further proposed strategies focus on increasing H3K9me3 at DSBs to modulate repair pathway choice. One hypothetical design is a Cas9‐SUV39H1 fusion, which might induce local H3K9me3 deposition and subsequent recruitment of HP1 and KAP1. Alternatively, it is hypothesized that a Cas9 fusion to SPIN1 could enhance TIP60 recruitment through a strengthened interaction with H3K9me3, thereby activating the TIP60–ATM signalling axis and promoting HDR. (c, d) Targeted suppression of NHEJ‐associated histone modifications to bias CRISPR‐induced DNA damage toward HDR. (c) Disruption of the H4K20me2‐dependent recruitment of 53BP1. One proposed strategy, the pharmacological inhibition of the H4K20 methyltransferases KMT5B/C, (e.g., A‐196) may reduce H4K20me2 levels and limit 53BP1 binding at DSBs. An alternative strategy involves a Cas9 fusion to dominant‐ dn53BP1 or other 53BP1 inhibitors, which is directly interfere with the 53BP1–H4K20me2 interaction, attenuating the NHEJ pathway. (d) Disrupting the H2AK15ub‐dependent recruitment of 53BP1. Another hypothetical strategy involves a Cas9 fusion to the BARD1 domain, which specifically recognizes H2AK15ub, may promote localized recruitment of BRCA1 at DSBs. This competitive binding displaces 53BP1 from H2AK15ub‐marked chromatin and shifts repair toward HDR. Alternatively, Cas9 fusion to dnRNF168 or transient inhibition of RNF168 (e.g., via siRNA) represents another conceptual approach, intended to reduce RNF168‐mediated H2AK15ub, thereby limiting NHEJ signalling and promoting HDR.

H3K36me3 also participates in cooperative chromatin networks with TIP60‐mediated H4K16 acetylation [102, 103]. We hypothesize that enhancing TIP60 localization at DSBs could potentially enhance H3K36me3‐based HDR. TIP60 is a mechanistically more attractive target than other H4K16 acetyltransferases like MOF, which appeal to have less pathway‐specific role in DSB repair [104, 105]. This has led to a proposal for novel fusion designs, such as those involving Cas9 and LEDGF or TIP60‐interacting domains; however, these designs are speculative and require precise optimization to preserve enzymatic function and gene‐editing efficiency (Figure 4a).

### 5.2 H3K9me3‐Based Strategies to Enhance HDR‐Mediated Knock‐In

A purely speculative and unconventional strategy for enhancing HDR involves modulating the transient enrichment of H3K9me3 around DSBs, a mark traditionally associated with gene silencing. Following DNA damage, SUV39H1‐mediated H3K9me3 deposition and its propagation via KAP1/HP1 complexes form a localized repressive chromatin domain that activates TIP60 and ATM signalling [74, 75]. This suggests creating transient and localized H3K9me3 levels might increase gene knock‐in efficiency. Translating this hypothesis into practice, however, remains a significant challenge, with several proposed yet to be validated approaches requiring careful consideration. For instance, one could propose directly depositing this mark by recruiting the SUV39H1 catalytic domain via Cas9. Similarly, given the recent research of the SPIN1‐H3K9me3‐TIP60 axis in DNA repair [76, 106], a future design might involve tethering SPIN1‐interacting domains to Cas9, which could stabilize H3K9me3 recognition. However, such protein‐tethering strategies carry intrinsic risks; even transient elevation of a protein with oncogenic potential like SPIN1 would demand safety assessment [107, 108]. An alternative putative approach is to use small‐molecule inhibitors to block the KDM4 family of H3K9me3 demethylases. However, global inhibition might lead to widespread H3K9 hypermethylation, chromatin compaction and consequently reduced Cas9 accessibility [99, 100]. Moreover, sustained high H3K9me3 levels may paradoxically hinder the inducible H3K9me3 surge at DSBs that is required for efficient end resection. Therefore, it is necessary to explore small molecule inhibitor concentrations and treatment times to regulate and balance H3K9me3 modification levels in euchromatin, and next‐generation sequencing technologies such as ChIP‐seq and ATAC‐seq can be used to detect global histone modification levels and chromatin accessibility after inhibitor removal (Figure 4b).

Overall, increasing H3K9me3 levels to increase gene knock‐in efficiency appears to be a double‐edged sword, as it may produce context‐dependent and even opposing outcomes. Thus, precise and locus‐specific regulation of H3K9me3 deposition remains a critical challenge that warrants systematic investigation and mechanistic clarification (Figure 4b).

### 5.3 H4K20me2‐ and H2AK15ub‐Based Strategies to Attenuate NHEJ and Enhance HDR‐Mediated Knock‐In

In addition to promoting HDR‐associated chromatin features, weakening NHEJ‐favour histone modifications represents a complementary strategy. This approach primarily targets the recruitment of the key NHEJ factor 53BP1, which recognizes H4K20me2 and H2AK15ub at DSB sites. Reducing these markers diminishes 53BP1 accumulation and shifts repair toward HDR.

H4K20 methylation is sequentially catalysed by KMT5A/B/C, and inhibition of these enzymes reduces 53BP1 foci formation while increasing reliance on HDR [109, 110, 111]. Small‐molecule inhibitors such as A‐196 have been shown to suppress NHEJ and facilitate HDR‐associated chromatin states, potentially through the replacement of H4K20me2 with TIP60‐mediated H4K20 acetylation [112]. Crucially, the application of A‐196 in gene editing has not been experimentally demonstrated. Furthermore, KMT5 inhibition is also associated with elevated γH2AX levels, suggesting potential genomic instability and careful safety evaluation (Figure 4c,d).

An alternative and more direct approach is to interfere with 53BP1 function itself. This has been successfully achieved using engineered inhibitors (e.g., i53), dominant‐negative 53BP1 variants, or competitive binding partners that block its interaction with H4K20me2 and H2AK15ub [54, 113]. H2AK15 ubiquitination, catalysed by RNF8‐RNF168, is another critical signal for 53BP1 recruitment. Inhibiting this pathway promotes TIP60‐dependent acetylation and shifts repair from NHEJ toward HDR [80]. Additionally, some exploratory strategies such as coupling Cas9 with to inhibitory peptides or BRCA1‐recruiting domains (likeBARD1 fragments) [114], might increase HDR at targeted loci, but lack experimental evidence. However, while the inhibition of 53BP1 recruitment represents a rational strategy to increase knock‐in efficiency, the pleiotropic role of 53BP1 in genome maintenance requires cautious implementation. Transient functional interference, although potentially beneficial at the target locus, may compromise repair fidelity at unrelated DSBs (Figure 4c,d).

### 5.4 Locus‐Specific Epigenetic Profiling to Enable Chromatin‐Context Gene Knock‐In Strategies

The choice of DNA repair pathway following a DSB is influenced by the surrounding chromatin features. In euchromatin, pre‐existing histone modifications, including acetylation (e.g., H3K14ac, H3K27ac, H4K16ac), methylation, and ubiquitination, and those induced post‐damage collectively influence DNA repair [115]. For example, pre‐existing H4K16ac at a DSB will promote HDR, and a local increase in H3K36me3 after a DSB in regions where H3K36me3 was not pre‐existing can enhance HDR [73, 116]. These changes in histone modifications on euchromatin provide a direction to specifically increase gene knock‐in efficiency. The epigenetic context is equally crucial within heterochromatin, which exhibits distinct functional domains. Regions marked by H3K27me3 preferentially utilize NHEJ, whereas those marked by H3K9me3 tend to favour HDR. Therefore, when targeting H3K27me3‐rich silent regions, a principle for adaptation would involve transiently erasing this repressive mark or converting the local environment to a more euchromatin‐like state prior to editing. Despite these insights, current research often lacks systematical epigenetic landscapes at specific target sites. Therefore, to effectively apply epigenetic regulation in precision genome editing, the focus should not be merely on histone modifications that mark general euchromatin or heterochromatin, but on the complex regulatory network of chromatin modifications at specific loci. Consequently, it is essential to construct comprehensive, high‐resolution chromatin maps capable of predicting locus‐specific repair outcomes. The technologies required for such site‐specific epigenetic profiling remain a challenging and developing field of research.

Beyond the technological challenge of epigenetic profiling, the practical application of epigenetic modulation faces another complexity. Histone modifications in DNA repair are dynamic and context‐dependent and require transient and spatially restricted epigenetic interventions to avoid global chromatin disruption. Future development requires systematic evaluation of chromatin accessibility, genome integrity, and transcriptional changes. Furthermore, large Cas9 fusion proteins may compromise delivery and stability, highlighting the need for minimalist, modular designs. High‐throughput computational screening and AI‐guided optimization may reduce early‐stage experimental complexity. Although the modulation of histone modifications offers a promising strategy to increase gene knock‐in efficiency, this approach remains challenging because of the complex chromatin networks regulating DSB repair pathway choice.

## 6 Challenges and Future Perspectives

Epigenetic regulation constitutes a critical and dynamic determinant of DSB repair pathway choice and, consequently, CRISPR–Cas9‐mediated knock‐in efficiency. Rather than serving as a passive substrate, chromatin acts as an active participant in coordinating histone modifications, nucleosome organization, and repair factor recruitment to influence DNA repair. Importantly, knock‐in efficiency does not scale linearly with chromatin accessibility; instead, repair outcomes reflect a coordinated interaction among the chromatin state, cell cycle, and pathway competition. Both preexisting and damage‐induced chromatin modifications influence HDR, providing a conceptual framework to elucidate variability in knock‐in efficiency beyond differences in nuclease activity.

Although modulation of histone modifications to increase knock‐in efficiency represents a promising strategy, its clinical and translation application presents significant challenges. However, these obstacles are facilitating innovative and constructive directions for future development.

### 6.1 Challenges

Global epigenetic modulation using small‐molecule inhibitors remains a convenient and effective method for rapid studies. However, its inherent non‐specificity can lead to transcriptional dysregulation and compromise genomic stability. As a more targeted alternative, targeting specific epigenetic modifiers or chromatin remodellers for knockdown via siRNA offers for more precise control over a particular class of modifications, thereby reducing the risk of global perturbations. Yet, achieving the goal of locus‐specific modulation, which involves modifying the chromatin environment exclusively at the target locus requires even more precise molecular tools. CRISPR‐based epigenome editing systems, such as Cas9 fusion proteins, are designed precisely for epigenetic modulation. However, their development is inherently challenging. First, the fusion of large epigenetic functional domains to Cas9 increases protein size, creating a significant hurdle for packaging and delivery via viral (e.g., AAV). Second, the design of these fusion proteins requires a delicate balance between functional potency, minimal steric hindrance, and the preservation of nuclease fidelity.

The efficacy of epigenetic strategies is highly dependent on the cellular context, including cell type and state. Interventions validated in rapidly proliferating cell lines often fail to translate directly to primary or quiescent cells, such as non‐dividing cells and haematopoietic stem cells (HSCs) [117]. This difference arises from fundamental differences in chromatin accessibility, DNA replication dynamics, and the predominant DNA repair pathways. Therefore, no single strategy, whether it involves small molecules, siRNA, or Cas9 fusions, can be expected to perform optimally across all cell types and genomic loci.

Any strategy aimed at increasing gene knock‐in efficiency, from global inhibitors to targeted Cas9 systems, must be validated by systematic safety evaluation. This is particularly critical for induced pluripotent stem cells (iPSCs) or highly plastic cells, where we suggest safety assessment as an indispensable component of strategy development. While the transient epigenetic modulation approaches discussed here are intended to mitigate long‐term risks, even transient alterations can interact with DNA repair processes, potentially increasing the risk of chromosomal rearrangements or unintended transcriptional changes. Consequently, establishing a comprehensive safety assessment guideline including karyotyping, whole‐genome sequencing for off‐target analysis, ATAC‐seq and ChIP‐seq for chromatin accessibility and related histone modification changes, and transcriptome profiling, which is an essential prerequisite for clinical translation.

### 6.2 Future Perspectives

To overcome these challenges, one promising research direction involves utilizing chromatin profiling to systematically map the epigenetic landscape of genomic loci. Separately, to advance the rational engineering of fusion proteins, AI and machine learning could integrate multi‐omics data (e.g., ATAC‐seq, ChIP‐seq) with performance data from existing functional domains. This would facilitate the *in silico* prediction and screening of novel fusion proteins optimized for maximum efficacy and safety within specific chromatin environments for personalized and context‐dependent editing strategies. Even when minimizing the fusion protein's size by using core functional domains, the construct often still surpasses the limited packaging capacity of AAV. This prompts the exploration of strategies focused on either engineering the Cas9 protein itself for a more compact size or developing alternative delivery platforms with larger payloads, such as exosomes or LNPs.

The application of epigenetic modulation is not only limited to increasing conventional CRISPR–Cas9‐mediated HDR efficiency, but also holds great synergistic potential with next‐generation editing tools. Prime Editing (PE) has revolutionized the precise insertion of large DNA fragments without inducing double‐strand breaks, thereby exhibiting a favourable safety profile with low off‐target effect [118, 119]. In non‐viral CAR‐T cell manufacturing, for example, PE has achieved impressive integration efficiencies (up to 50%) for a 3 kb CAR cassette in primary human T cells with minimal toxicity, establishing it as a transformative platform for therapeutic T‐cell engineering [120]. However, other studies result also show that PE efficiency differs across different local chromatin environments. For instance, knock‐in efficiency is significantly higher at the accessible *TRAC* locus compared to the more compact *AAVS1* locus [118]. This result clearly highlights the synergistic potential of combining transient epigenetic modulation with precise gene editors. A promising future direction involves transient epigenetic modulation to create a more permissive chromatin environment, and to employ a high‐fidelity editor like PE for efficient and precise integration. This combination strategy enables consistent, efficient, and safe large‐scale gene knock‐in across a broader range of genomic loci and cell types, representing a major innovative development for CRISPR‐mediated precision genome engineering.

## Author Contributions

Xin Chen and Zichuan Liu contributed to conception and design, and all drafted and revised the manuscript. Xueren Li, Yanhua Gong, and Zichuan Liu critically read and revised the manuscript.

## Funding

This work was supported by the National Key R&D Program of China (2024YFA0916600), Tianjin University Key Project of the Graduate Top‐notch Innovation Award Program in Arts and Sciences (C1‐2024‐010), Jinnan Hospital Research program (JNYYKY‐202301), and the Funding of Thinkingbiomed Biotechnology (Beijing) Co. Ltd. (2022GKF‐0269).

## Conflicts of Interest

The authors declare no conflicts of interest.

## Data Availability

Data sharing not applicable to this article as no datasets were generated or analysed during the current study.
